# Spatial overlap of shark nursery areas and the salmon farming industry influences the trophic ecology of *Squalus acanthias* on the southern coast of Chile

**DOI:** 10.1002/ece3.2957

**Published:** 2017-04-18

**Authors:** Juan Diego Gaitán‐Espitia, Daniela Gómez, Alistair J. Hobday, Ross Daley, Julio Lamilla, Leyla Cárdenas

**Affiliations:** ^1^ CSIRO Oceans and AtmosphereHobartTASAustralia; ^2^ Facultad de CienciasInstituto de Ciencias Ambientales y EvolutivasUniversidad Austral de ChileValdiviaChile; ^3^ Institute for Marine and Antarctic StudiesUniversity of TasmaniaHobartTASAustralia; ^4^ Facultad de CienciasInstituto de Ciencias Marinas y LimnologicasUniversidad Austral de ChileValdiviaChile

**Keywords:** artisanal fishery, reproduction, Salmon industry, sexual maturity, *Squalus acanthias*, trophic ecology

## Abstract

Potential interactions between marine predators and humans arise in the southern coast of Chile where predator feeding and reproduction sites overlap with fisheries and aquaculture. Here, we assess the potential effects of intensive salmon aquaculture on food habits, growth, and reproduction of a common predator, the spiny dogfish—identified as *Squalus acanthias* via genetic barcoding. A total of 102 (89 females and 13 males) individuals were collected during winter and summer of 2013–2014 from the Chiloé Sea where salmon aquaculture activities are concentrated. The low frequency of males in our study suggests spatial segregation of sex, while immature and mature females spatially overlapped in both seasons. Female spiny dogfish showed a functional specialist behavior as indicated by the small number of prey items and the relative high importance of the austral hake and salmon pellets in the diet. Immature sharks fed more on pellets and anchovies than the larger hake‐preferring mature females. Our results also indicate that spiny dogfish switch prey (anchovy to hake) to take advantage of seasonal changes in prey availability. Despite differences in the trophic patterns of *S. acanthias* due to the spatial association with intensive salmon farming, in this region, there appears to be no difference in fecundity or size at maturity compared to other populations. Although no demographic effects were detected, we suggest that a range of additional factors should be considered before concluding that intensive aquaculture does not have any impact on these marine predators.

## 1 Introduction

In the past three decades, salmonid aquaculture in Chile has experienced an explosive growth and diversification driven by the use of intensive farming techniques (Buschmann et al., 2009; Oddone, Paesch, & Norbis, 2015) and the introduction of several different salmonid species (Correa & Gross, 2008). Although this growth has placed Chile as the second most important salmon exporter worldwide (Ellis et al., 2016), it has also raised criticisms and concerns related to its negative environmental effects (Buschmann et al., 2006, 2009; Soto & Norambuena, 2004). The marine phase of salmon aquaculture is conducted in an open water net‐system that allows the discharge of large quantities of pollutants, mainly organic matter, nitrogen, and phosphorus into water bodies and the sea floor (Paudel et al., 2015). Discharges from salmon farms can lead to eutrophication and alterations of the seasonal nutrient supply of coastal ecosystems (Brooks & Mahnken, 2003; Kutti et al., 2007) and the sediment organic content (Carroll et al., 2003; Soto & Norambuena, 2004), producing zones of anoxic conditions (Brown, Gowen, & McLusky, 1987; Buschmann et al., 2006). All of these environmental changes around salmon farms impact the structure and composition of benthic communities by supporting high production of deposit‐feeding invertebrates (Guilpart et al., 2012; Kutti et al., 2007) and attracting high densities of demersal fish (Carss, 1990; Dempster et al., 2002) and other mobile carnivores (Bonizzoni et al., 2014; Kemper et al., 2003; Quick, Middlemas, & Armstrong, 2004; Ribeiro et al., 2007; Sepúlveda & Oliva, 2005; Veríssimo, McDowell, & Graves, 2010). The immediate outcome of these alterations is enhancement of benthic‐pelagic coupling and the potential transfer of sediment‐associated contaminants into higher trophic levels (Debruyn et al., 2006).

**Figure 1:**
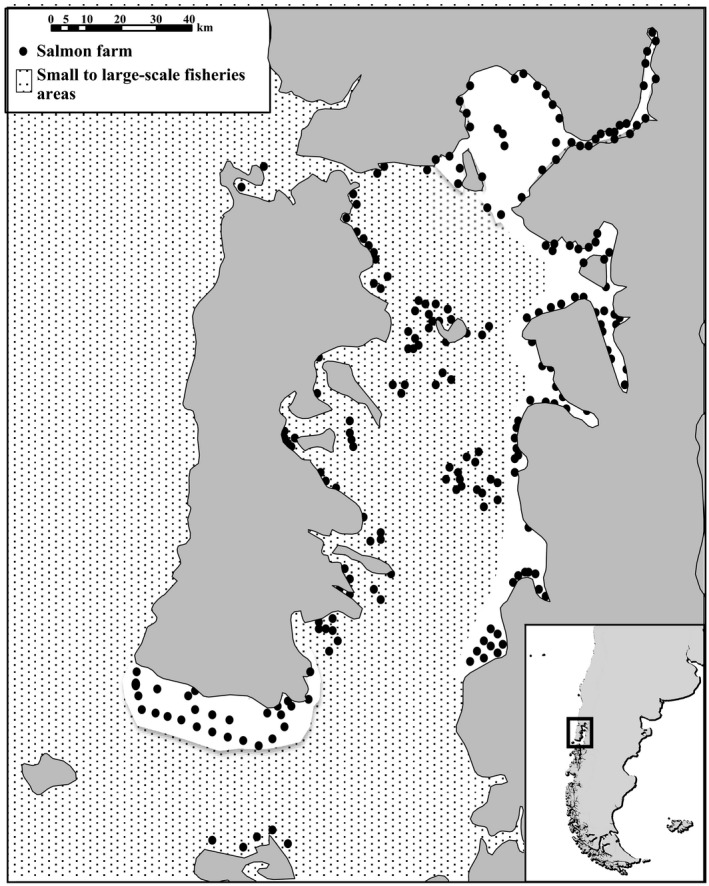
Map of the study area with salmon farms distribution and activity areas of small to large‐scale fisheries (e.g., hake, anchovies, mackerel, cusk‐eel, sea urchins) (modified from Bustos‐Gallardo & Irarrazaval, 2016)

In Chile, the salmon industry is concentrated around the inner sea of Chiloé Island (41–43°S, hereafter Chiloé Sea) (Figure 1) (Buschmann et al., 2006, 2009). This region is a semi‐closed environment that is home to rare, unique and endangered marine species, including cold‐water corals (Försterra & Häussermann, 2003), penguins and migratory birds (Senner et al., 2014; Skewgar, Boersma, & Simeone, 2014), otters (Ebensperger & Botto‐Mahan, 1997), blue and humpback whales (Hucke‐Gaete et al., 2004), dolphins and pinnipeds (Ribeiro et al., 2007; Veríssimo et al., 2010), and skates and sharks (Lamilla et al., 2005; Quiroz, Wiff, & Céspedes, 2009; Valenzuela, Bustamante, & Lamilla, 2008). Most of these marine animals use the Chiloé Sea as a feeding and nursery area (Hucke‐Gaete et al., 2004; Quiroz et al., 2009). However, this semi‐closed environment is under great pressure because the high density of human‐related activities that are carried out in this region has become a major threat for the ecological balance and persistence of local marine species (Oddone et al., 2015). For instance, different biological aspects (e.g., mortality rates, behavior and distribution) of a range of marine predators, such as seals, dolphins, and sharks, are significantly affected by their interactions with fisheries (e.g., hake, mackerel, sardine, and anchovy) and intensive aquaculture systems (e.g., salmon, mussels) in the Chiloé Sea (De La Torriente et al., 2010; Kemper et al., 2003; Ribeiro et al., 2007; Sepúlveda & Oliva, 2005; Veríssimo et al., 2010). Some of these interactions have led to mortalities as a consequence of direct human attack or by accidental bycatch (De La Torriente et al., 2010; Ribeiro et al., 2007; Sepulveda et al., 2007). One of the best examples of this situation is the spiny dogfish *Squalus acanthias*. This coastal squaloid shark, as well as other dogfish species (e.g., *Centroscyllium granulatum* and *Deania calcea*), is common bycatch species in many small and large‐scale fisheries in southern Chile (De La Torriente et al., 2010; Lamilla et al., 2005; Valenzuela et al., 2008). Unfortunately, little is known about the abundance of *S. acanthias* in Chile and the ecological implications of the population declines (~30%) documented for this vulnerable species in South America (Fordham et al., 2006; Veríssimo et al., 2010).

Given the potential for strong interactions between human activities (aquaculture and fisheries) and spiny dogfish populations in southern Chile (De La Torriente et al., 2010; Valenzuela et al., 2008), here we assessed the potential effects of salmon farming on the biology of *S. acanthias* in the Chiloé Sea. We also discuss the trophic and reproductive metrics employed here in the context of *S. acanthias* populations elsewhere, which are unexposed to intense salmonid aquaculture.

## 2 Material and Methods

Spiny dogfish (*N* = 102; 89 females and 13 males) were collected from bycatch of artisanal fisheries in the inner sea of Chiloe Island (41°40′S; 72°39′W) at the northern part of the Chilean Southern fiords area (Figure 1) during May–August (winter) 2013 and December–February (summer) 2014. These fisheries exploit small pelagic fish species such as jack mackerel, anchoveta, hake, sardine among others. Once captured, spiny dogfish specimens were preliminarily identified by morphological examination, preserved in ice, and transported to the Elasmobranches Laboratory at the Austral University of Chile (UACh). Gender and weight (*W*) were recorded, while maturity stage was determined following Stehmann (2002). Precaudal length (PC_L_) and total length (*T*_L_) were measured to the nearest gram and millimeter for each animal.

### 2.1 Species identification

Many bycatch species of dogfish are difficult to identify owing to extensive morphological similarities. This identification problem is exacerbated by the common fishery practice of removing the head, tail, and most fins from landed sharks while still at sea to reduce required storage space for the animal. In order to confirm the identity of the spiny dogfish species captured by the artisanal fishery in the inner sea of Chiloé, and assumed to be *S. acanthias*, we sequenced a 670‐bp fragment of the cytochrome c oxidase subunit 1 gene (*Cox1*). Total genomic DNA (gDNA) was extracted from muscle of 10 randomly selected individuals using the standard phenol‐chloroform method. *Cox1* sequences were amplified by polymerase chain reaction (PCR) using primers reported elsewhere (Fallabella, 1994; Ward et al., 2007). PCR products were purified using the ENZA DNA Purification Kit (OMEGA). Sequencing was performed at the Departamento de Ecología—Universidad Católica de Chile. All sequences were compared against the GenBank nonredundant protein database using BLASTN. Sequences showing significant hits (*E* value < .001) and high percent of identity (>99%) were downloaded from GenBank for posterior analysis. Sequences were aligned using the MAFFT platform of the TranslatorX multiple sequence alignment program (Abascal, Zardoya, & Telford, 2010). Alignments were performed using the L‐INS‐i option (accurate for alignment of ≤200 sequences) and default settings. A median‐joining haplotype network was constructed with the PopART program (Leigh & Bryant, 2015) to estimate the “fine scaled” intraspecific relationships among Chilean sequences and the reference sequences obtained from GenBank. Haplotype network analyses are powerful methods for intraspecific data in revealing multiple connections between haplotypes and the number of base‐pair changes between them (Mardulyn, 2012).

### 2.2 Trophic ecology

To investigate how diet around the salmon cages may vary with shark size, specimens were divided into two classes—mature or immature, following size cutoffs provided in Alonso et al. (2002). Stomachs were preserved in 10% formalin and stored frozen at −20°C until analyzed. Stomach contents were thawed and washed with water. Identification was based on intact and remaining hard structures combined with general shape and anatomical features of prey. Recognizable prey items were identified to the lowest possible taxon, following the osteological guide of fishes (Fallabella, 1994) from the Biological Collection at the Zoology Institute of the UACh. Because the sample size was relatively small, cumulative number of prey curves was used to evaluate adequacy of the sample size in our study. These curves were estimated with asymptotic models using two different approaches: (1) the rarefaction curve and (2) the trophic diversity curve based on the number of prey items and the number of stomachs sampled (Cortés, 1997; Mardulyn, 2012). The rarefaction curve was generated by means of the EstimateS 8.2 software (Colwell, 2013), randomizing the sample order 100 times. Thereafter, the asymptotic Clench function was fitted to the smoothed curve *S*(*e*) = *ae*/(1 + *be*), where *S*(*e*) is the number of species found per sampling‐effort unit (*e*); *a* and *b* are function parameters. The latter were adjusted to the data of each curve by means of a Simplex and Quasi Newton method of the Statistica package (StatSoft Inc. version 6.0, 2004) to calculate regression coefficients and asymptote. The composition and relative importance of food items in the diet were examined using the three indices described by Hyslop (1980): (1) the numerical index (%*N*); (2) the frequency of occurrence (%*O*); and (3) the gravimetric index (%*W*). If used separately, these indices can produce a bias toward highly abundant items (%*O*), small items (%*N*), or very large items (%*W*) (Mardulyn, 2012). To overcome these individual biases, a generalized form of the relative importance index (%RI) was calculated to determine the combined effect of these indices on each prey category (Vögler, Milessi, & Duarte, 2009). The %RI was plotted to establish the level of importance of each prey category according to discontinuities in the slope of the curve. The categories of prey were sorted according to level of importance where high %RI values were assigned to the first level of importance and so forth (Vögler et al., 2009). Finally, diet breadth was calculated using Levin's standardized index, ranging from 0 to 1, where low values indicate diets dominated by few prey items (specialist predators), and high values indicate generalist diets (Krebs, 1989).

For the data set, a similarity matrix was generated using the Bray–Curtis similarity measure. In addition, the nonparametric analysis of similarities (ANOSIM) was run on the matrices using 999 permutations with the PRIMER software (v 6.0). We tested the null hypothesis of no differences in the diet composition between the mature and immature female sharks, and between winter and summer (grouping maturity stages). Percent data were square‐root‐transformed prior to statistical analysis, which satisfied assumptions of normality.

### 2.3 Reproductive biology

Reproduction of *S. acanthias* in the Chiloé Sea may also be influenced by association with salmon aquaculture. Because of the low number of males collected in the area of study, we focused investigation on the 89 females. A regression analysis was first conducted to determine length–weight relationships using the following equation:

$$W=a\times L^{b}c$$

where *W* is the weight (g), *a* and *b* are constants, and *L* is the total length in cm.

The presence of mature yellow oocytes (diameter over 2 cm) and embryos within the uteri was considered to indicate mature females (Stehmann, 2002). Relationships between mother size and the number of eggs and embryos were then determined. Total length at 50% maturity (*T*_L50%_) was calculated by a logistic model fitted to the maturity data as follows:

$$Y=\left[1+e-\left(a+b\times T_{L}\right)\right]-1$$

where *Y* is the proportion of mature individuals and *T*_L50%_ is given by—*a*/*b* (Nakano, Hayashi, & Nagamine, 2015). Litter size was recorded for pregnant females, along with sex, total length (*T*_Lpup_), weight (*W*_pup_), and volume of the yolk sac (*V*) for each embryo. Relationships between length–weight, length–volume of the yolk sac, and the sex ratio of the embryos were explored using the lm() function in the R statistical package. The developmental stage of the embryos was estimated following Jones and Ugland (2001). A two‐way ANOVA was performed for comparison of the number of embryos among maturity classes and seasons.

## 3 Results

### 3.1 Species validation

Spiny dogfish samples (*n* = 10) from the Chiloé Sea were successfully amplified, sequenced, and deposited at the NCBI under the accession numbers GU191146 to GU191155. Comparison of these sequences against GenBank showed high similarity (>99%) to *S. acanthias* followed by *S. suckleyi* (98%), *S. edmundsi* (93%), and *S. hemipinnis* (92%) (Fig. S1). Consequently, sequences of *S. acanthias* from different parts of the world were used as reference in the median‐joining haplotype network (Fig. S2). Only one haplotype was shared among populations in the Southern (Chile, Argentina, Tasmania, South Africa) and the Northern (USA, Canada, Europe) Hemisphere. All other haplotypes are inferred to have been derived from this haplotype by one (e.g., Chilean and Argentinian populations) or >2 mutational steps (e.g., UK and USA populations) (Fig. S2).

### 3.2 Diet composition

**Figure 2:**
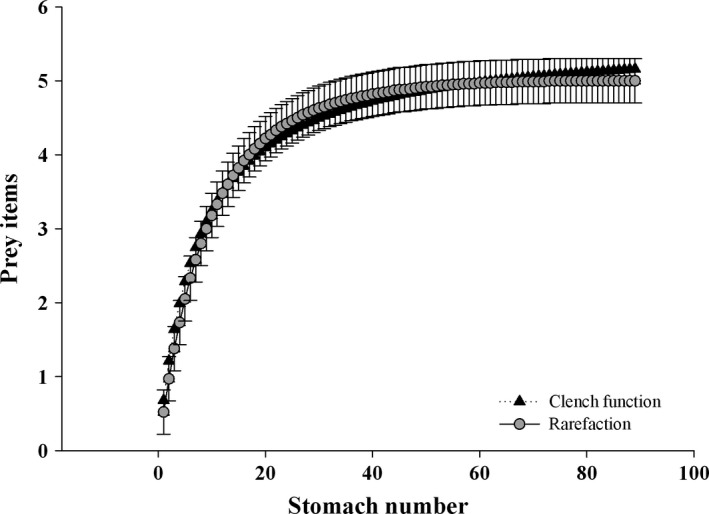
Cumulative prey curves for females of *Squalus acanthias*, the vertical axis shows the number of prey items and the horizontal axis shows the number of guts analyzed

**Figure 3:**
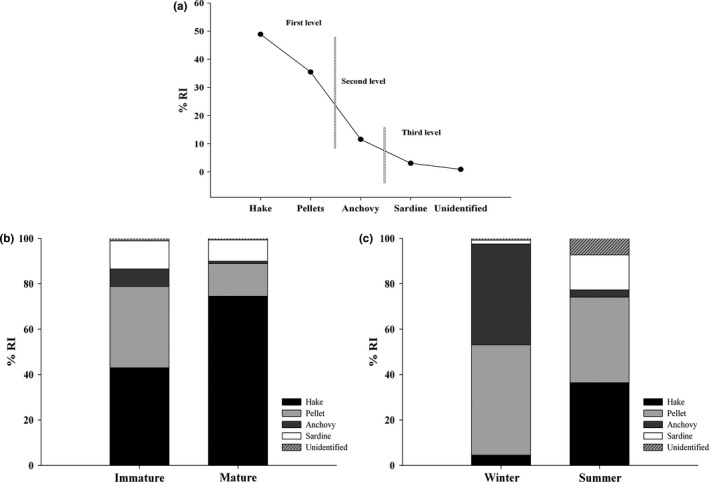
Trophic ecology in females of *Squalus acanthias*. (a) Relative importance (%RI) of functional prey categories. Vertical lines indicate the three levels of importance of the functional prey categories according to discontinuities in the slope of the %RI curve. Comparative analysis of the relative importance (%RI) between (b) maturity stages and (c) seasons

Stomach contents of 26 immature (range 46–74 cm *T*_L_; mean ± 1 *SD*: 57 ± 8.1 cm) and 63 mature (71–98 cm *T*_L_; 85 ± 5.8 cm) females, were examined, with 61 (68.5%) stomachs containing food items. Whole and undigested prey items were frequently found in the same stomachs as well‐digested prey. The rarefaction curve and the trophic diversity curve showed a well‐defined asymptote (Figure 2), indicating that the total sample size was adequate in describing spiny dogfish diet in the Chiloé Sea. The stomach content of female sharks was composed of five prey items (Table 1), where fish were the most represented taxa with three items: austral hake (*Merluccius australis*), anchovy (*Engraulis ringens*), and sardine (*Sprattus fuegensis*). *M. australis* was the dominant prey, contributing the highest values of trophic indices followed by food pellets from salmon farms (Table 1). According to the %RI, the diet of female spiny dogfish is characterized by three levels of importance (Figure 3a), where hake and pellets are grouped in the first level, anchovy in the second level, and the third level is comprised of sardine and unidentified prey items (e.g., gelatinous prey). The Levin's standardized index (Ba) indicated a narrow trophic niche breath (Ba = 0.31) for *S. acanthias* in the Chiloé Sea as a result of the low number of prey items and the higher importance of austral hake in the diet of female sharks. The relative importance of pellets for immature females decreased in the diet of mature sharks, in part due to increased %RI of the austral hake (Table 1, Figure 3b). However, the results of the ANOSIM test indicated that the diets of these two groups were not significantly different (Global *R* = .008, *P* = .399). Comparison of stomach contents during austral seasons revealed some differences in the %RI between winter and summer (Global *R* = .165, *P* = .044; Table 1, Figure 3c). For instance, during winter, anchovy and salmon pellets represent 93% of the prey contribution to the %RI, while in summer, hake and pellets are the most important items with ~74% of RI (Table 1, Figure 3c). No differences in the number of immature and mature females were detected between seasons (*χ* ^2 ^= 1.9, *P* = .59).

**Table 1:** Diet composition of females *Squalus acanthias*

	Total			Stages of development			Seasons	
Stomach contents	%*O*	%*N*	%*W*	%RI	%RI mature	%RI immature	%RI winter	%RI summer
Unidentified	13.1	5.2	1.5	0.93	0.7	1.1	0.8	7.3
Pellet	57.4	30.9	26.6	35.5	14.4	35.8	48.6	37.7
Hake	49.2	40.4	52.4	48.9	74.5	43.0	4.5	36.4
Anchovy	37.7	15.7	13.1	11.6	1.0	7.7	44.4	3.2
Sardine	19.6	7.8	6.5	3.1	9.4	12.4	1.7	15.4

### 3.3 Reproductive development and fecundity

**Figure 4:**
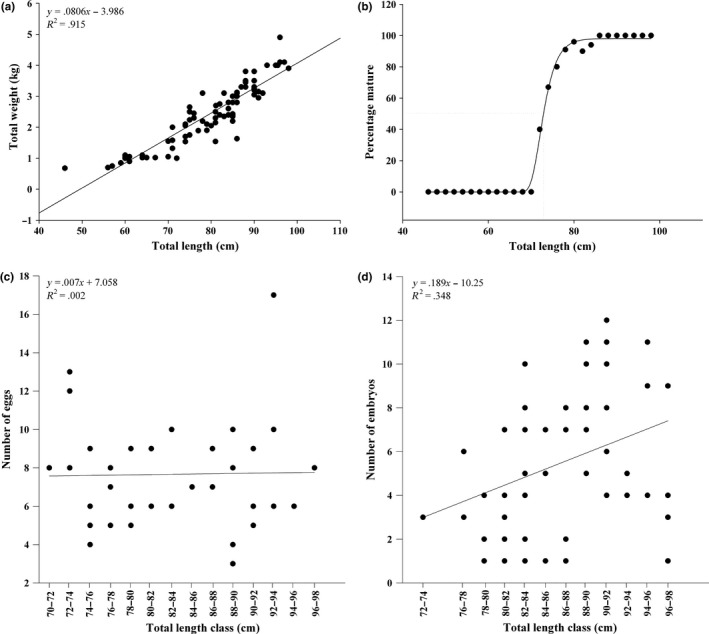
Reproductive biology of *Squalus acanthias* females in the Chiloe Sea. (a) Allometric relationship between the total weight and total length of spiny dogfish. Relationships between length of female sharks and (b) the percentage of mature females, (c) the number of eggs and (d) the number of embryos

**Figure 5:**
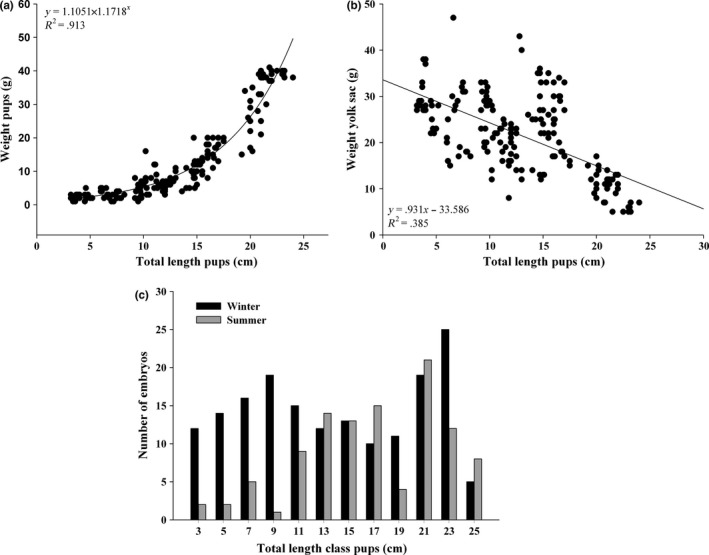
Relationship between the total lengths of pups with (a) their body and (b) yolk sac weights. (c) Changes in distribution of the total lengths of pups between summer and winter

Females of *S. acanthias* collected in the Chiloé Sea ranged in length from 46 to 98 cm (mean ± 1 *SD*: 79 ± 11 cm). Their weights varied between 0.7 and 4.9 kg (mean ± 1 *SD*: 2.4 ± 1 kg). The relationship between these two variables revealed a positive allometric trend for somatic growth (Figure 4a, *R* ^2^ = .915; *P* < .001). The estimated length at 50% maturity was 72.8 cm (*r* = .998, Figure 4b). The number of oocytes with a diameter over 2 cm varied between three and 17 (mean ± *SD*: 7.67 ± 2.8 oocytes), and no significant relationship was detected between the *T*_L_ of mature females and the number of eggs (Figure 4c, *R* ^2^ = .002; *P* < .91). On the other hand, the number of embryos showed a low but positive relationship with the *T*_L_ of the female sharks (Figure 4d, *R* ^2^ = .348; *P* < 0.012) ranging between one and 12 embryos per female (mean ± *SD*: 5.52 ± 3.1). A total of 274 embryos were found with lengths between 3.6 and 25.8 cm (mean ± *SD*: 12.7 ± 5.7 cm) and weights between 2.5 and 40.8 g (mean ± *SD*: 12.3 ± 11.9 g). The relationship between total length (*T*_Lpup_) and weight (*W*_pup_) of pups suggests that embryos increase in weight exponentially (Figure 5a, *R* ^2^ = .913; *P* < 0.001) and as they grow the weight of their yolk sac decreases (Figure 5b, *R* ^2^ = .385; *P* < 0.001). The sex ratio of the embryos was even (1:1), and significant differences were detected in the number of embryos at the smaller mature size classes between seasons (Figure 5c, two‐way ANOVA; *F*_10,251_ = 1.96, *P* = .038).

## 4 Discussion

Interactions between marine predators and humans arise in coastal ecosystems in various parts of the world where feeding and reproduction sites overlap with economic activities such as fisheries and aquaculture. These interactions include (1) mortality through bycatch; (2) deliberate harming of animals by humans; (3) changes in animal behavior; (4) competition for the same resource; (5) changes in prey size structure and distribution; and (6) changes in community composition resulting from fishing or from aquaculture‐induced alterations of the environment (De La Torriente et al., 2010; Guilpart et al., 2012; Kemper et al., 2003; Kutti et al., 2007; Papastamatiou et al., 2010; Ribeiro et al., 2007; Sepúlveda & Oliva, 2005; Sepulveda et al., 2007; Veríssimo et al., 2010). Given the high density of human‐related activities (e.g., fisheries and intensive aquaculture systems) in the Chiloé Sea (Oddone et al., 2015), strong interactions with spiny dogfish populations (De La Torriente et al., 2010; Valenzuela et al., 2008) were possible. Our results show that the spatial overlap of shark nursery areas and the salmon industry influences the trophic niche of *S. acanthias* in this region by adding new food items (i.e., pellets; direct effect), and altering the structure and composition of benthic communities (e.g., crustaceans; indirect effect).

### 4.1 Genetic validation of *Squalus acanthias*

Although sharks are not the target species in many Chilean fisheries, practices such as drift netting and long line fishing result in high levels of shark bycatch (Lamilla et al., 2005; Valenzuela et al., 2008). The accurate identification of species of sharks obtained from bycatch is difficult because some species differ only slightly in their morphology and also because in some cases, head, tail, and most fins from landed sharks are removed at sea to minimize storage needs and prevent spoiling (Shivji et al., 2002). Therefore, different molecular approaches have been recommended for shark species identification. One approach is DNA barcoding using universal primers to amplify part of target genes (Holmes, Steinke, & Ward, 2009; Ward et al., 2008). This approach is similar to the traditional morphology‐based methods, where species identification is based on the presence or absence of a distinct morphological feature (Viswambharan et al., 2015). In our study, the use of the cytochrome c oxidase subunit 1 gene (*Cox1*) as a barcoding sequence allowed us for the first time to unambiguously identify *S. acanthias* as the dogfish species in the bycatch of artisanal fisheries in the region. As with other reported barcoding studies, the *Cox1* region is effective in delineating sharks up to the species level within the genus *Squalus* (Ward et al., 2007). Despite the relatively low number of individuals sequenced in this study, we were able to identify 10 closely related haplotypes in these Chilean dogfish. One of these haplotypes was shared mainly with other *S. acanthias* populations in the Southern Hemisphere. These results are consistent with a previous global phylogeographic study, in which the South Pacific and Atlantic lineage of *S. acanthias* was characterized by a star‐shaped network with one very common, central haplotype and several derived and low‐frequency haplotypes (Veríssimo et al., 2010).

### 4.2 Trophic ecology of *Squalus acanthias* around salmon farms in Chile

The overall diet of *S. acanthias* females in the Chiloé Sea consisted mainly of teleost fishes. However, commercial salmon feeds were present in the stomachs of female dogfish. This is the first record of this high‐energy food item (24–30% oil, ~19 kJ g^−1^) in *S. acanthias*, which differs from the natural trophic composition for this species in other regions of the world (Alonso et al., 2002; Avsar, 2001; Demirhan, Seyhan, & Basusta, 2007; Dunn et al., 2013; Hanchet, 1991; Holden, 1966; Laptikhovsky, Arkhipkin, & Henderson, 2001; Tanasichuk et al., 1991). Salmon farms are known to affect the surrounding environment and local marine communities by the addition of organic matter in the form of feces and uneaten feed (Kutti et al., 2007; Soto & Norambuena, 2004). Organic waste from salmon cages produces a high concentration of ammonia in the local environment that can lead to decreased growth, increased vulnerability to diseases and pathological changes in animals inhabiting salmon farming areas (Piedecausa et al., 2010). Here, the presence of salmon pellets in the diet of *S. acanthias* raises concern about the potential impact of salmon farms on the biology of marine predators, their trophic relationships, and ecosystem function in this region. For example, the use of corn or soybean in commercial pellets provides a high proportion of oleic acid (18:1n‐9), linoleic acid (18:2n‐6) and linolenic acid (18:3n‐3), that alter the fatty acid composition, physiology and behavior of fish and mussels from areas in the vicinity of salmon farms (Redmond et al., 2010; Skog et al., 2003). In addition, the intensive nature of salmon farming has resulted in the use of antibiotics (e.g., oxytetracycline), to limit bacterial and protozoan infections (Primavera, 2006). These antibiotics are administered with the feed pellets and have the potential to be accumulated in tissues of marine organisms around the salmon cages (Campbell, Pantazis, & Kelly, 2001). Excessive use of antibiotics results in the development of side effects, such as liver damage, toxic effects, bacterial resistance, and immune suppression, and can have negative effects on environmental quality and human health (Nakano et al., 2015; Zounková et al., 2011). In Chile, salmon aquaculture uses 8000 times more antibiotics than in other countries (e.g., Norway), and the presence of tetracyclines (used for protein inhibition) and quinolones (fast bactericide action) has been documented in native species surrounding the salmon pens of the Chiloé Sea (Buschmann et al., 2009; Outeiro & Villasante, 2013; Soto & Norambuena, 2004).

Beyond the presence of salmon pellets in the diet of *S. acanthias* in Chile, the trophic ecology of this squaloid differs compared to populations in other regions. Overall, spiny dogfish are described as a fairly non‐selective (i.e., generalist) and opportunistic predator that feeds on many pelagic and benthic species (Alonso et al., 2002; Belleggia et al., 2012). However, female spiny dogfish in Chile showed a functional specialist behavior as indicated by the small number of prey items and the relative high importance of the austral hake and pellets in the diet. The trophic niche of *S. acanthias* populations in the Northern Hemisphere, the Southwest Atlantic, and Southwest Pacific is characterized by a great diversity of teleost fishes (e.g., hakes, sandeels, gobies, herrings, anchovies, and whiting), crustaceans (e.g., shrimps, crabs, lobsters, and euphausiids), mollusks (e.g., squids and octopuses), and other invertebrates (e.g., salps, sea anemones, nematodes, and polychaetes) (Alonso et al., 2002; Avsar, 2001; Belleggia et al., 2012; Demirhan et al., 2007; Dunn et al., 2013; Hanchet, 1991; Holden, 1966; Laptikhovsky et al., 2001; Tanasichuk et al., 1991). In most of these studies, demersal and pelagic fish were the main component of the diet, which is consistent with our result. However, in these regions, *S. acanthias* feeds on other important prey items such as crustaceans and mollusks that are absent in the diet of sharks in the Chiloé Sea. One plausible explanation of this discrepancy could be the low occurrence of these preys near salmon farms in Southern Chile. In fact, one of the most drastic environmental effects of organic waste from salmon aquaculture is the alteration of the structure and composition of benthic communities (Guilpart et al., 2012; Kutti et al., 2007) and particularly the reductions in biodiversity of crustaceans in the vicinity of salmon cages (Buschmann et al., 2009; Hall‐Spencer et al., 2006; Outeiro & Villasante, 2013).

From an ontogenetic perspective, *S. acanthias* exhibits substantial variation in diet with body length (e.g., Alonso et al., 2002; Avsar, 2001; Demirhan et al., 2007; Dunn et al., 2013; Hanchet, 1991). This is consistent with our results in which smaller immature sharks fed more on pellets and anchovies than larger mature females. The ontogenetic transition from small prey items (e.g., crustaceans, salps, other invertebrates) to fish feeding is a typical feature of the trophic ecology of *S. acanthias* (Dunn et al., 2013; Tanasichuk et al., 1991). Another source of variation in the diet composition of the spiny dogfish is seasonality (Belleggia et al., 2012; Demirhan et al., 2007; Laptikhovsky et al., 2001; Tanasichuk et al., 1991). Here, despite of the presence of salmon pellets in the stomachs of female sharks throughout the year, significant differences were detected between summer and winter, again consistent with other studies that show spiny dogfish switch prey to take advantage of spatiotemporal changes in prey availability (Demirhan et al., 2007). In the Chiloé Sea, sharks prey more on anchovies during winter and hakes during summer, which is consistent with the local availability of these prey fishes due to their seasonal migration (Aguayo‐Hernández, 1995; Leal et al., 2011).

### 4.3 Biological parameters

The relatively larger size and increased residence time of fishes near salmon farms compared with fishes from other areas is explained by the availability of high‐energy pellets from salmon cages, as documented in many species elsewhere (Skog et al., 2003). Hence, differences in biological parameters for spiny dogfish in the Chiloé Sea compared to populations not influenced by salmonid aquaculture might be expected. However, our results did not support the hypothesis of different reproductive and growth patterns in spiny dogfish in Chiloe. The size range of immature and mature females was similar to southwest Atlantic populations (Alonso et al., 2002; Nakano et al., 2015), and slightly lower than Northern Hemisphere populations (Avsar, 2001; Gračan et al., 2016; Jones & Ugland, 2001; Yigin & Ismen, 2013), but with positive allometric trends for somatic growth (*T*_L _~ *W*) in all of the cases. The size at 50% maturity of *S. acanthias* in the Chiloé Sea (72.8 cm) was higher than reported for the Mediterranean Sea (51.8–52.8 cm) (Chatzispyrou & Megalofonou, 2005; Yigin & Ismen, 2013), similar to Southwest Atlantic (70.4 cm) (Nakano et al., 2015) and Southwest Pacific (71.5–74.0 cm) populations (Hanchet, 1988), and lower than in the Black Sea (88 cm) (Avsar, 2001), the Northeast (77–78.2 cm) (Henderson, Flannery, & Dunne, 2002; Stenberg, 2005) and Northwest (79.1 cm) (Sosebee, 2005) Atlantic, and British Columbia waters (93.5 cm) (Ketchen, 1972). Although regional differences in *T*_L50%_ occur, there is no evidence of particular effects of local conditions (i.e., salmon farming) on this parameter of *S. acanthias* in the Chiloé Sea. Fecundity (i.e., number of eggs and embryos) was also within the values described for *S. acanthias* elsewhere in the world, with an increasing number of embryos in larger mature females, and no differences in their sex ratio (Avsar, 2001; Jones & Ugland, 2001; Ketchen, 1972; Nakano et al., 2015). The range of total length of embryos examined encompasses the theoretical curve proposed by Avsar (2001) for this species and follows the relationship for embryo weight described in other regions (Chatzispyrou & Megalofonou, 2005; Yigin & Ismen, 2013). The low frequency of males in our study suggests that spatial segregation (latitude and depth) of sex may occur, while immature and mature females spatially overlapped in all seasons (Nakano et al., 2015).

Overall, despite differences in the trophic patterns in this region, from this study, there appears to be no demographic expression in terms of fecundity or size at maturity of these trophic changes due to the spatial association with intensive salmon farming. We have not calculated the calorific value of the diet, and we suggest that this variable is needed in one additional comparative study with samples from other regions that are not influenced by salmon farming, to test any diet enrichment hypothesis. It may also be necessary to examine the effect of any chemicals in the environment on the reproductive performance of the sharks. If this inner sea is the only dogfish nursery area in the region, then a reproductive comparison may not be possible, and global comparisons of dietary energy and reproductive output will be needed. We conclude that at this stage, the intensive salmon aquaculture of the Chiloé Sea is influencing the diet of *S. acanthias*, but without an impact on shark reproduction.

## Conflict of interest

None declared.

## Author contribution

LC, JDGE, DG, and JL conceived and designed the study. DG sampled the biological material and carried out DNA extractions. JD, DG, AH, and RD performed data analyses. JD, AH, and LC drafted the manuscript. All authors read, approved, and contributed to the final manuscript.
