# Supplementary figures and images for: Spatial overlap of shark nursery areas and the salmon farming industry influences the trophic ecology of Squalus acanthias on the southern coast of Chile
Source: Ecol Evol. 2017 Apr 18;7(11):3773–83. doi: 10.1002/ece3.2957 (PMC5468132; doi:10.1002/ece3.2957)

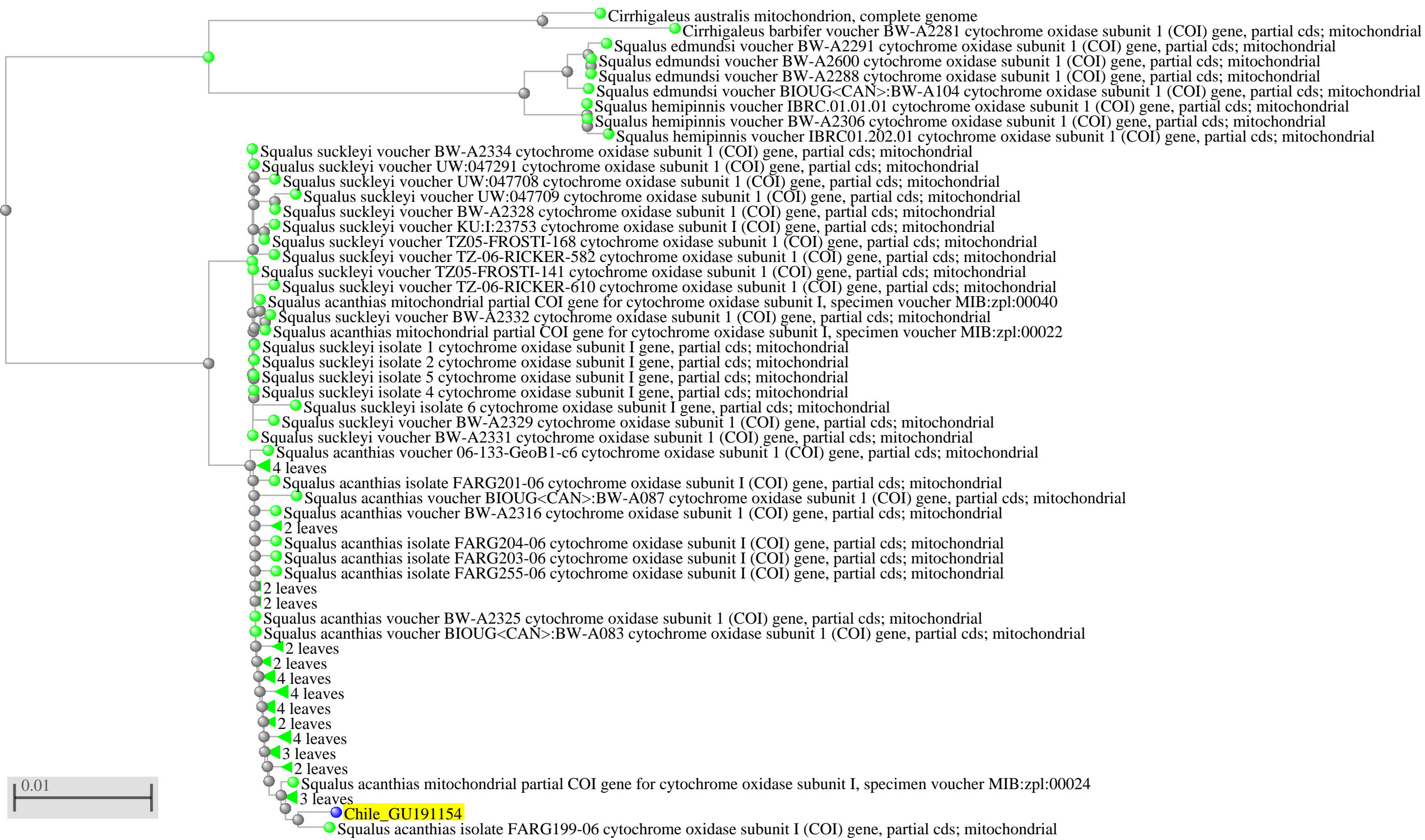

Supplement: Supplementary file 1 [file ECE3-7-3773-s001.pdf]

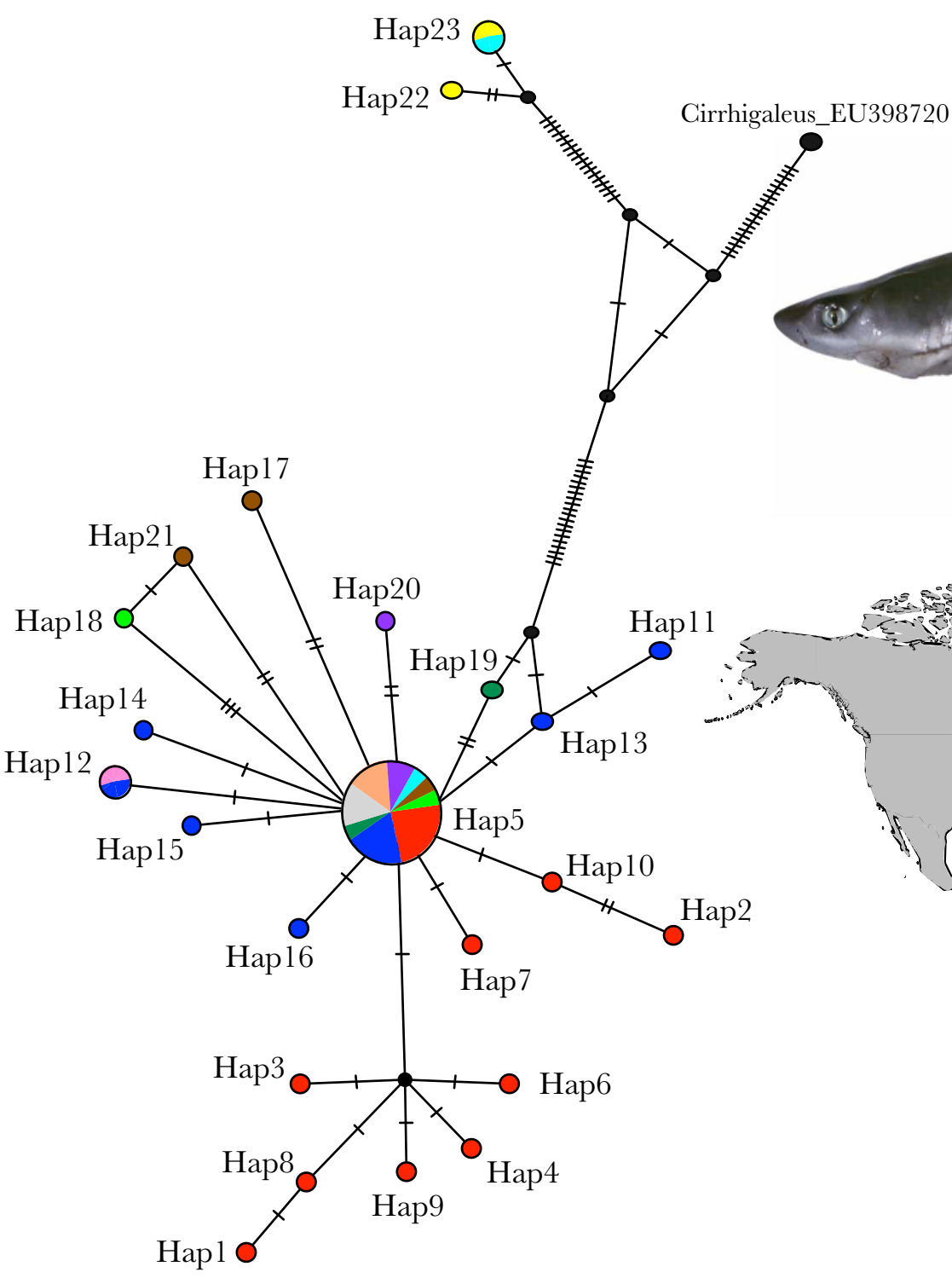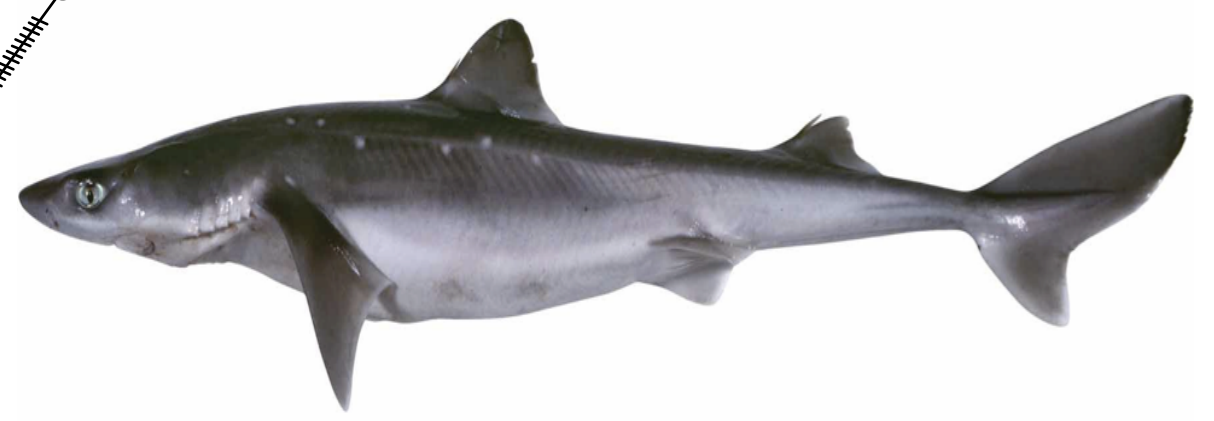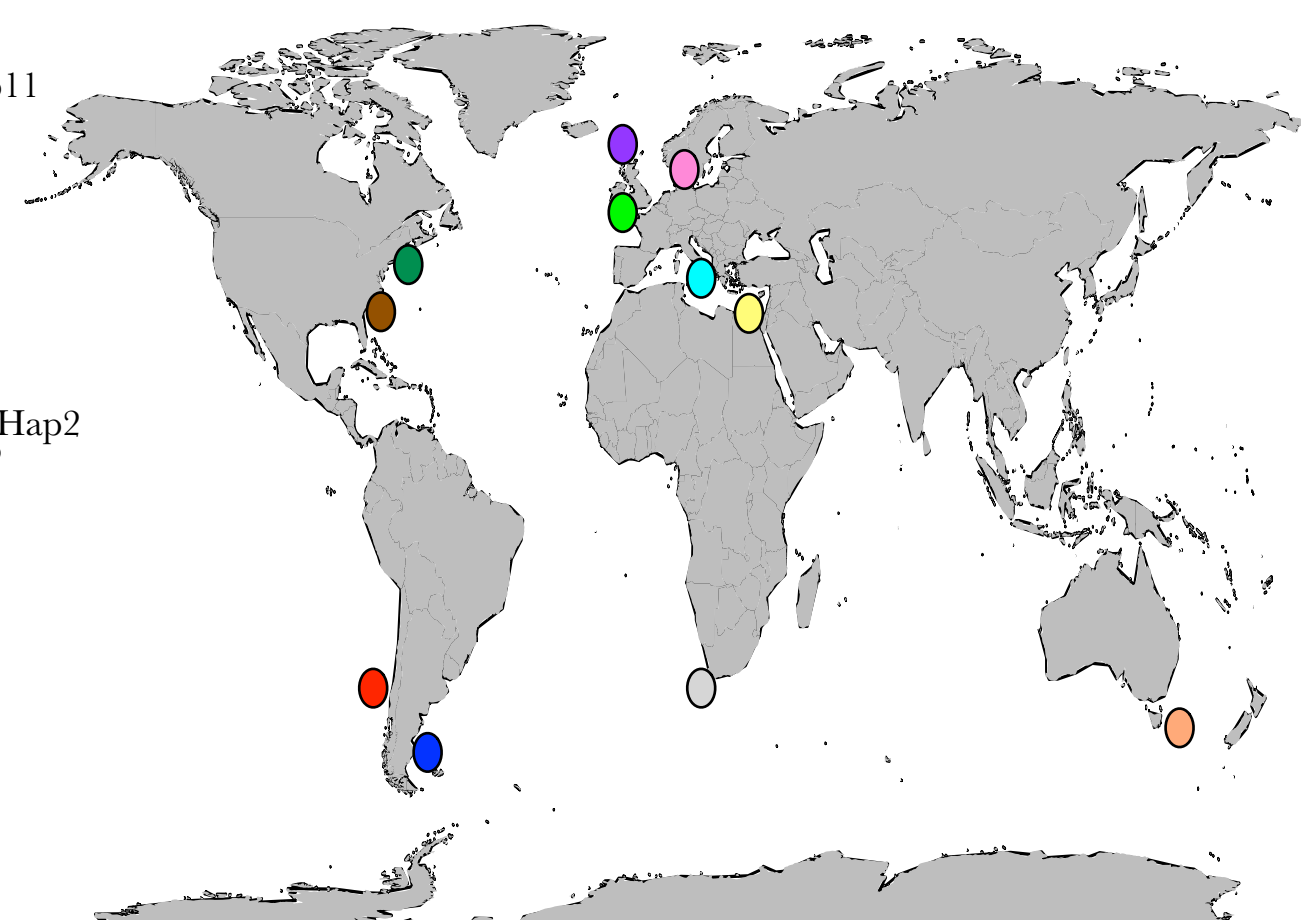

Supplement: Supplementary file 2 [file ECE3-7-3773-s002.pdf]
